# A cost-effective laboratory device for single slow micro compression testing of soft materials in the small-strain region

**DOI:** 10.1016/j.ohx.2025.e00738

**Published:** 2025-12-28

**Authors:** Jaehyeong Kim, Sangjun Pyo, Hyerin Ahn, Ok Chan Jeong

**Affiliations:** a Department of Digital Anti-aging Healthcare, Inje University, Gimhae 50834, the Republic of Korea; b Department of Biomedical Engineering, Inje University, Gimhae 50834, the Republic of Korea

**Keywords:** Micro-compression testing, Mechanical properties, Cost-effective instrumentation, Small strain region, Laboratory devices

## Abstract

In various microscale applications, accurate evaluation of the mechanical properties of materials under small-strain and low-force conditions is important. However, conventional universal testing machines (UTM) are expensive and difficult to operate reliably under low-force conditions, making them unsuitable for small-strain testing. To overcome these limitations, we developed a cost-effective device for single slow micro-compression testing (MCT) to measure the mechanical properties of materials in the small-strain region. The MCT consists of a force sensor (FlexiForce A301-1, Tekscan, USA), an Arduino-based signal acquisition module, and a high-precision z-stage. The displacement control accuracy of the z-stage was verified using a laser displacement meter (LK-G10, Keyence, Japan), and the experimental results confirmed displacement and force resolutions of 1 µm and 0.01 N, respectively. Under no-load conditions, the force–displacement error between the MCT and a commercial universal testing machine (MTS, AMETEK LRX-plus, LLOYD INSTRUMENTS, UK) was within ± 2.0 %. In addition, standard and one-third-size PDMS (polydimethylsiloxane) specimens were fabricated according to ASTM D575-91 and tested using both systems; the error between the two specimens was within ± 0.05 %. From the linear region in the small-strain range, the Young’s modulus of the miniature specimen was estimated, showing a percentage error of + 2.2 % from reported values, confirming high precision and reliability.

The developed device, with a total fabrication cost below USD 1,500, provides cost-effectiveness, precision, and repeatability, enabling applications in polymer MEMS and soft robotics.

## Highlights

• Developed a low-cost micro compression tester for small-strain region.
• Achieved high resolution: 1 µm displacement and 0.01 N force detection accuracy.
• Verified ± 2.0 % agreement with commercial MTS through force–displacement comparison.
• Performed compression tests with standard PDMS specimens (ASTM D575-91)
• Easy-to-fabricate and cost-effective system broadly applicable in MEMS research.

## Graphical abstract

**Figure:**
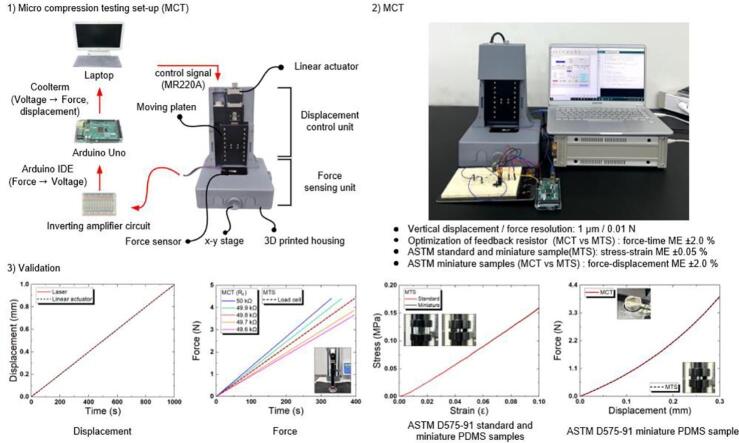

## 1 Specifications table

.Table 1.Table 2.Table 3.

**Table:** 

Hardware name	Micro Compression Testing set-up (MCT)
Subject area	• Engineering and materials science
Hardware type	• Material testing
Closest commercial analog	• MTS (LLOYD INSTRUMENTS, AMETEK LRX-plus) • TA Instruments Electro Force 3200 Series • Instron 5940 Series Universal Testing Systems*.”*
Opensource license	CC-BY-SA-4.0
Cost of hardware	1485.53
Source file repository	https://doi.org/10.5281/zenodo.17337730

**Table 1:** Summary of design files.

Design file name	File type	Open-source license	Location of the file
FlexiForce_A301_ datasheet	PDF	Not applicable	https://www.mouser.kr/datasheet/2/1460/FLX_Datasheet_A301_RevI-3084724.pdf
Inverting amplifier circuit	PCB schematic	CC-BY-4.0	Available with the article
Arduino_board	PDF (Pinout Diagram)	MIT License	https://store.arduino.cc/products/arduino-mega-2560-rev3?srsltid = AfmBOoqZnD6UMg8t-Lrt4OUPb0fxuTX-8XtxbUWEzDRjs94GgzhkbqAD
Arduino_force_sensor _code	Software	CC-BY-4.0	Available with the article
SMX-8060-X_Motor_Stage_technical_drawing	Specifications	Not applicable	https://st1.kr/shop/item.php?it_id = 1669784728
SMX-8060 X _Motor_Specifications	Specifications	Not applicable	https://st1.kr/shop/item.php?it_id = 1669784728
Housing	CAD	CC-BY-4.0	https://doi.org/10.5281/zenodo.17337730

**Table 2:** Summary of bill of materials.

Designator	Component	Number	Cost per unit ($)	Total cost ($)	Source of materials	Material type
R_S_	FlexiForce A301-1 Sensor	1	12.22	12.22	https://www.mouser.kr/ProductDetail/Tekscan/A301-1?qs = 8Wlm6%252BaMh8RdzjLjoiBU0Q%3D%3D	Electronic components
PCB	E-CALL EIC Breadboard	1	22.22	22.22	https://www.coupang.com/vp/products/1365664487	Composite
R1 − R4	100 kΩ Resistor	4	0.97	3.88	https://www.mouser.kr/ProductDetail/TE-Connectivity-Holsworthy/ROX5SJ100K?qs = s1WWODT2SXVoSGv%252B%2FYqWYg%3D%3D	Electronic components
A1	Arduino Uno Board	1	50.23	50.23	https://store.arduino.cc/products/arduino-mega-2560-rev3?srsltid = AfmBOoqZnD6UMg8t-Lrt4OUPb0fxuTX-8XtxbUWEzDRjs94GgzhkbqAD	Composite
R_F_ (Variable resistance)	500 kΩ Potentiometer	1	1.73	1.73	https://www.mouser.kr/ProductDetail/BI-Technologies-TT-Electronics/P160KN-0QC15B500K?qs=%252BUYXD5bnyXqrvTnJjhEsOw%3D%3D	Electronic components
M1	MCP6004 Operational Amplifier	1	0.66	0.66	https://www.digikey.kr/ko/products/detail/microchip-technology/MCP6004-E-P/683200	Semiconductor
C_F_	47 pF Capacitor (500 V, 2 %)	1	2.05	2.05	https://www.mouser.kr/ProductDetail/Cornell-Dubilier-CDE/CD15ED470GO3F?qs = FKrQhPEeH%252B7YSlXXQw49cg%3D%3D	Electronic components
JW1	PCB Jumper Wire M/F	12	0.25	3.00	https://www.eleparts.co.kr/goods/view?no = 10996688	Electronic components
Z-stage	SMX-8060-X Motor controller and Stage	1	1371.55	1371.55	https://st1.kr/shop/item.php?it_id = 1669784728	Metal
U1	USB 3.2	1	17.99	17.99	https://www.amazon.com/SanDisk-Extreme-Type-Flash-Drive/dp/B08KSJ144R?th = 1	other

**Table 3:** Summary of MCT Validation and Measurement Accuracy.

Validation	Specimen	Equipment	Results and Accuracy
Validation of the vertical displacement of the z-axis linear actuator	−	Laser displacement meter	±0.1 %
Optimization of R_F_	−	MTS, MCT	49.8 kΩ selected ±2.0 %
Applicability of miniature specimens	Standard and miniature	MTS	+0.05 %
Validation of MCT efficiency	Miniature	MTS, MCT	±2.0 %
Young’s modulus estimation	Miniature	MCT	+2.2 %

## 2 Hardware in context

Microelectromechanical systems (MEMS) are widely used in sensors, actuators, biomedical structures, and microfluid systems [1], [2], [3], [4], [5], [6], [7], [8]. Most MEMS devices usually operate in a small displacement range, ranging from several micrometers to tens of micrometers [9], [10], [11], [12]. For example, the membranes of pressure sensors and force sensors, or micromirrors, produce a limited displacement response to external stimulations, which determines structural resistance and stability [13], [14], [15], [16], [17], [18]. The mechanical behavior of MEMS devices is usually controlled by a small force of several N to several mN. Consequently, a test system that can simultaneously consider both displacement and forces is necessary [19], [20], [21], [22], [23], [24]. It has been reported that soft actuators made of flexible materials, such as PDMS (polydimethylsiloxane) or electro-active polymers (EAPs), can measure ten to hundreds of displacements [25], [26], [27], but the operating force also often remains within the N–mN range [28], [29]. When measuring micro equipment with conventional equipment designed for the range of macro forces and displacements, the overall efficiency decreases due to the high cost of the equipment and the long measurement time required. In addition to MEMS applications, micro-scale compression tests are crucial to characterization of soft and compliant materials such as polymers, biological tissues, and liquid systems, and accurate measurements of low-force mechanical responses are essential. Therefore, it is essential to use custom test equipment that accurately measures behavior under small-scale conditions [30], [31], [32], [33], [34], [35]. The mechanical properties of hard materials such as silicon are studied by nanoindentation [36], [37], [38], loading tests [39], [40], microbeam bending [41], [42], resonance frequency analysis [43], [44] and digital image correlation (DIC) [45], [46]. However, these methods focus primarily on specific device deformation and their accuracy is greatly influenced by modeling assumptions and boundary conditions [47], [48], [49], [50]. Meanwhile, PDMS is widely used in flexible electronics and bioMEMS due to its flexible mechanical properties and excellent biocompatibility [51], [52], [53], [54], [55], [56], [57]. However, because of the high viscoelasticity and nonlinear stress–strain behavior, the accurate measurement of Young's modulus is still a difficult task [58], [59], [60]. Although existing commercial test equipment is very precise, its high cost and large system size limit the accessibility [61], [62]. In the context of MEMS applications, measurements of mechanical properties of micro displacement and microforce regions are more useful than other data. Full-range measurements using commercial equipment are not only inefficient but may also limit research at the laboratory level [63], [64], [65]. In recent years, small-scale testing and manufacturing devices based on low-cost open-source methods have been developed to overcome these limitations [66], [67], [68], [69]. Examples include the planar biaxial tension system [70], [71], the automatic divider system [72], [73], the photo tracking system [74], [75] based on Raspberry Pi, and the open source and 3D printed device [76], [77]. Gunter et al. [78] developed portable tensile testers to assess the hyper-elastic properties of biomaterials. Hinge et al. [79] It integrates optical extension meters into cheap desktop stress testers to accurately measure flexible polymer deformations. Woods et al. [80] presented open-source flexible material tension testers for educational and research environments. These devices analyze the mechanical behavior of flexible materials in laboratories, but focus on tensile testing. As a result, the need for compression testing devices has consistently increased [81], [82], [83]. Although small-scale devices are useful to measure material mechanical behavior in laboratories, they often use specimens in non-standard forms and make it difficult to obtain internationally recognized standards values [84], [85], [86]. The manufacture and evaluation of ASTM standard specimens is considered essential to clearly define the intrinsic properties of the material [87], [88], [89]. If small samples are used, their validity must be rigorously demonstrated by comparison with standard samples [90], [91], [92], [93]. The tensile tests of viscoelastic materials such as PDMS (ASTM D412, rubber and elastomer tensile tests) are widely conducted, but the results of the tensile and compression tests differ considerably [55], [94], [95], [96].

As a result, the compression test was performed on the basis of ASTM D575-91(Standard Rubber Compression Testing) to evaluate the properties of the material under conditions similar to those of the MEMS operating environment, to minimize the tightness of the viscoelastic and geometric nonlinearity in the small load region. The standard PDMS specimen and the 1/3-scale specimen are manufactured according to ASTM D575-91, and the tests are conducted using the proposed equipment and commercial equipment. Comparing the force displacement characteristics measured by both devices validates the applicability of the proposed device to scale samples and enables the estimation of Young's compressive modulus in the small strain region. In this study, compact and cost-effective MCT were developed to measure the compression mechanical properties of microstructures under low strain conditions. Unlike previous studies that focused on large-scale tensile testing, this study develops a low-cost, high-precision, single-stage slow micro compression device. The proposed system allows precise measurement of the mechanical properties of materials in small-scale and low-load areas and can be easily applied at laboratory level.

## 3 Hardware description

The proposed MCT consists of hardware and software modules. The hardware assembly comprises a linear actuator for vertical displacement control based on a z-stage (precision automatic linear X stage, SMX-8060-X, Sciencetown, Korea), a moving platen, a force sensor, an inverting amplifier circuit using an MCP6004 (Microchip Technology Inc., USA) operational amplifier, an Arduino Uno (Arduino, Italy), and a force measurement module connected to a laptop interface. The software assembly comprises the MR220A (NOVA Electronics, Japan) motor control software, the Arduino IDE (Arduino, Italy) for converting voltage to force, and CoolTerm (Roger Meier, Switzerland) for simultaneously storing force–displacement data.

### 3.1 The 3D-printed housing and frame

**Fig. 1:**
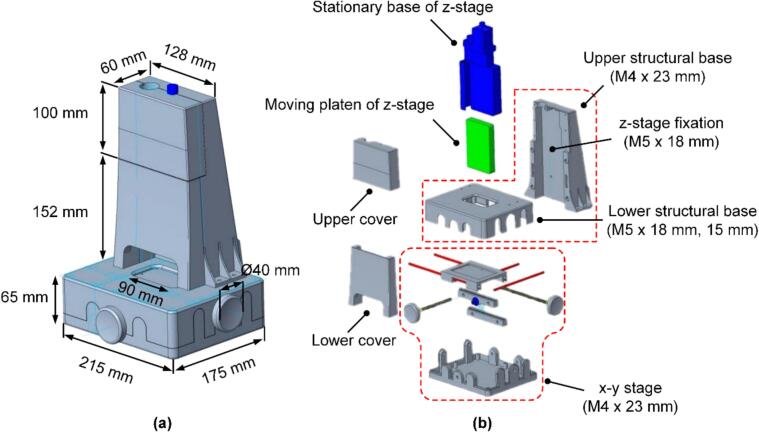
Captured images of the 3D-printed housing. Schematic view (a), exploded view (b).

Fig. 1 shows both a schematic and an exploded view of the 3D-printed housing. The structural base of the MCT serves as the primary supporting frame, ensuring mechanical stability and alignment of all mounted units. The upper and lower covers are detachable, allowing easy assembly, maintenance, and secure housing of the internal circuits. At the top, the stationary base of the z-stage serves as a rigid anchor point for vertical movement, and the moving platen travels along the compression axis to transmit a load to the specimen. At the bottom, an x-y stage enables precise horizontal positioning of specimens, ensuring proper alignment with the compression axis. All mechanical components were fastened using standard metric bolts. The z-stage and upper structural base were joined using M5 × 18 mm bolts, while the upper and lower structural bases were securely fastened using both M5 × 18 mm and M5 × 15 mm bolts. Additionally, M4 × 23 mm bolts were used to fasten the x-y stage to the structural base. This mechanical design, which combines bidirectional translation of the x-y stage with smooth vertical displacement of the moving platen, enhances placement accuracy and test repeatability. The housing was fabricated using an FDM 3D printer (Creality K2 Plus, 0.4-mm nozzle) with PLA filament. The total print time for the housing was approximately 14 h and 42 min, consuming 259.48 m (773.9 g) of PLA filament with a total cost of USD 15.48. Printing was performed using a 0.4-mm nozzle at a layer height of 0.2 mm and 30 % infill density with automatic support generation. The final height of the structure was approximately 278.4 mm.

### 3.2 Core mechanical and electronic components

**Fig. 2:**
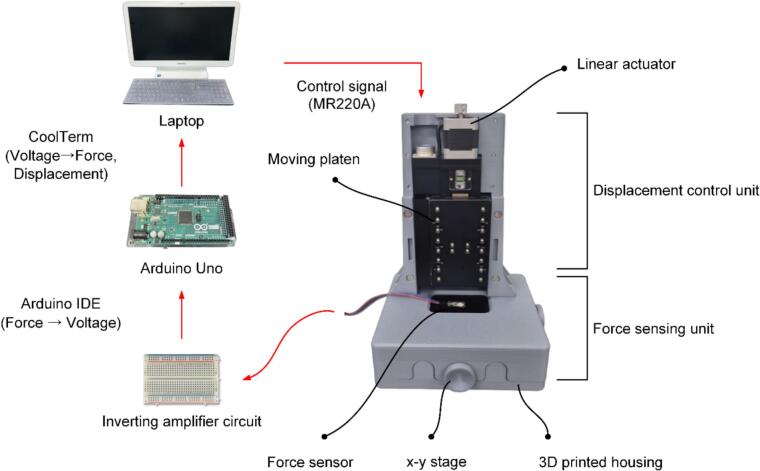
Schematic view of the proposed MCT for compression test.

Fig. 2 shows a schematic diagram of the developed Micro-Compression Tester (MCT). The proposed MCT consists of two parts: a hardware module and a software module. The hardware module is housed inside a 3D-printed enclosure, and the overall device configuration comprises a displacement-control unit and a force-sensing unit. The displacement control unit is based on a z-stage (precision automatic linear X-stage, SMX-8060-X, Sciencetown, Korea). Originally designed for horizontal (x-axis) displacement control, it was modified and used as a z-stage in this study to achieve precise vertical (z-axis) displacement. The z-stage's linear actuator drives a moving platen to generate uniaxial compression on the sample. During load application, the platen's movement can be controlled in micrometer increments. The force-sensing unit consists of a force sensor and an inverting amplifier circuit. This hardware assembly is compactly housed within a 3D-printed enclosure and secured to the base plate using M4 bolts to minimize errors caused by vibration and noise.

Specifications for the MCT components fabricated in this study are as follows: the force sensor has a sensing area with a diameter of 9.53 mm and can measure up to 4.4 N. The sensor's accuracy is 3 %, with linearity within ± 3 % of full scale, repeatability within ± 2.5 %, and hysteresis within ± 4.5 %. The response time is under 5 μs, and durability has been confirmed for over 3 million cycles. The operating temperature range is − 40 °C to 60 °C, and the temperature sensitivity is 0.36 %/°C. The sensor output is a voltage signal, and the connection method uses a solder-tab and pin structure. An Arduino Uno board based on the ATmega2560 microcontroller was used. The operating voltage is 5 V, and the operating clock speed is 16 Mhz. The z-stage employs a cross-roller guide to ensure high straightness and precision. It is driven by a feeding screw, with an overall position accuracy of ± 12 μm. Additionally, position-control stability was enhanced by including one home sensor and two limit sensors. The laser displacement meter used for displacement verification has a reference measurement distance of 10 mm and a measurement range of ± 1 mm. Its repeatability is 0.02 μm (maximum 0.01 μm).

### 3.3 System control and data acquisition

Control and data acquisition for this system were performed based on real-time communication between the hardware module and the software module. The voltage measured by the force sensor is amplified through the MCP6004 operational amplifier. When an external load is applied, the force measured by the sensor is stored in the Arduino as a voltage signal, and the voltage is converted into a force value using the Arduino IDE software. Meanwhile, the vertical displacement data of the z-stage are acquired through the input signal of the MR220A motor control software. Input pulses set in the MR220A drive the linear actuator, and the vertical displacement calculated based on the pulse period is recorded. The measured displacement-force data are stored via a laptop using CoolTerm software. During post-processing, a force–displacement curve is generated to enable real-time monitoring of measurements and ensure consistency of test data.

## 4 Design files summary

### 4.1 FlexiForce_A301_data sheet

This file contains the technical datasheet for the FlexiForce A301 sensor, including the specifications, operating parameters, and usage guidelines.

### 4.2 Inverting amplifier circuit

This is an inverting amplifier circuit design that integrates the force sensor with other components, allowing accurate measurement of force signals.

### 4.3 Arduino_board

This file includes a pinout diagram for the Arduino board and details the connections required for interfacing with external components.

### 4.4 Arduino_force_sensor_code

This software file includes the Arduino code used to read voltage data and convert the data into force measurements.

### 4.5 SMX-8060-X_Motor_Stage_technical_drawing

This technical drawing provides the detailed dimensions and mechanical specifications of the SMX-8060-X motor stage. These are essential for integration and mounting.

### 4.6 SMX-8060-X_Motor_Stage_Specifications

This file lists the specifications of the SMX-8060-X motor stage, including the resolution, speed, accuracy, and load capacity.

## 5 Bill of materials summary

### 5.1 Build instructions

#### 5.1.1 Assembly steps for housing and z-stage

**Fig. 3:**
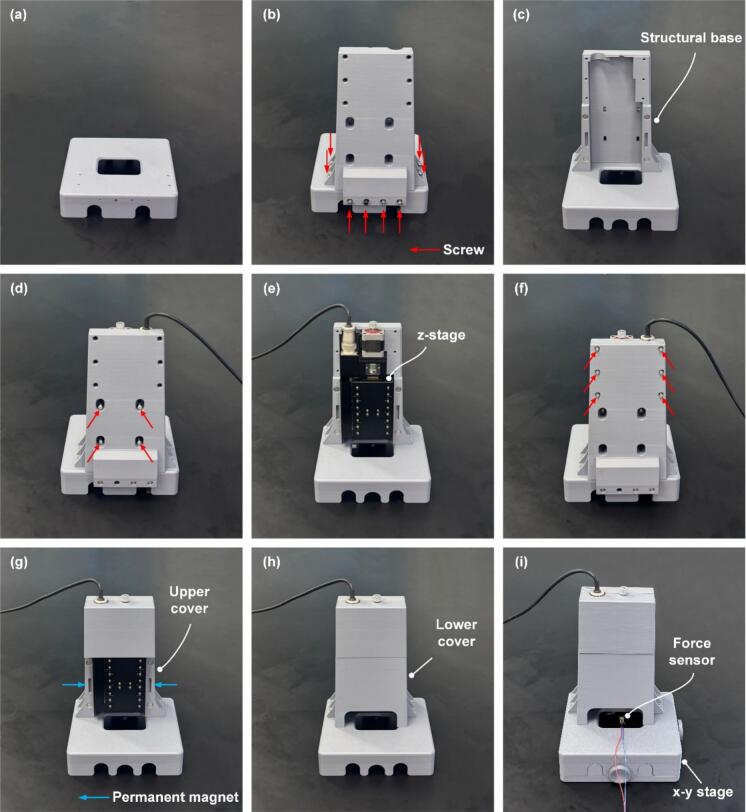
Assembly steps of housing and z-stage. Place the structural base (a), assemble the lower and upper parts of the structural base and tighten the screws (b, c), mount the z-stage, which serves as the driving component, onto the structural base (d), secure the z-stage by fastening the screws on the front side (e), attach the upper cover to the front top of the z-stage and fasten with screws (f). assemble the lower cover to the bottom of the structural base (h), attach the x–y stage, with the force sensor mounted at its center, to the lower part of the structural base to complete the assembly (i).

Fig. 3 illustrates the assembly steps of the 3D-printed housing and the high-precision z-stage using a linear actuator. First, place the lower part of the structural base on a flat surface (Fig. 3a). Next, align the lower and upper parts of the structural base and fasten them evenly with screws (Fig. 3, Fig. 3). Then, assemble the z-stage onto the structural base and secure it with screws (Fig. 3, Fig. 3). At this point, ensure that the moving platen faces forward and is aligned with the central axis of the x–y stage to be attached later. Attach the upper cover to the top front of the z-stage and tighten it (Fig. 3f). Install an N40-grade neodymium–iron–boron (NdFeB) permanent magnet between the z-stage and the structural base to facilitate the attachment of the lower cover and to enhance positional stability by minimizing structural instability during compression (Fig. 3g). Mount the lower cover to the front bottom (Fig. 3h). Finally, attach the x–y stage to the lower part of the structural base to complete the assembly (Fig. 3i). The force sensor is mounted at the center of the x–y stage.

#### 5.1.2 Electrical circuit

**Fig. 4:**
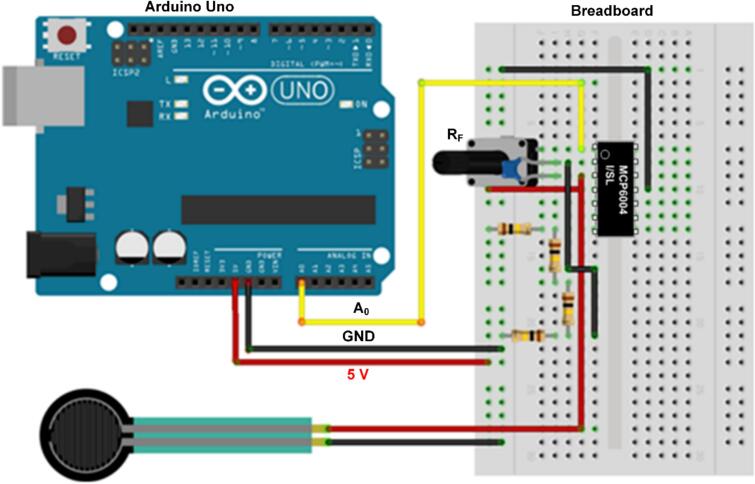
Pin configuration for connecting the inverting amplifier circuit to the Arduino.

Fig. 4 shows the pin configuration for connecting the force-sensing inverting amplifier circuit to the Arduino. The circuit consists of a force sensor, operational amplifier, variable feedback resistor, capacitor, and power supply. The operational amplifier is configured as an inverting amplifier, where the resistance of the force sensor is connected between a fixed reference voltage and the inverting input (−), while the non-inverting input (+) is connected to ground. The variable feedback resistor (R_F_) and capacitor are connected in parallel. The circuit output is connected to the Arduino input terminal (A_O_)**,** and the Arduino is linked to the laptop via a USB cable to complete the MCT system.

**Fig. 5:**
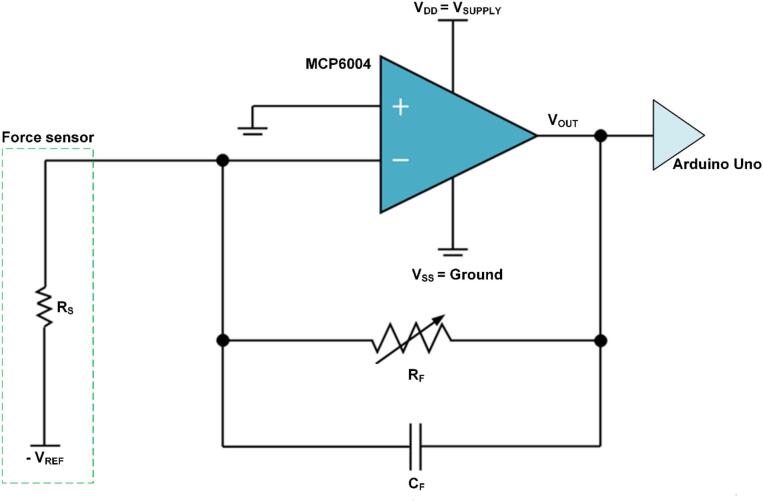
Equivalent electrical model of the force sensor and inverting amplifier circuit for force-to-voltage conversion. The output voltage (V_OUT_) was calculated as − V_REF_ multiplied by the gain defined by the ratio between the variable resistance (R_S_), which changes with external pressure, and the variable feedback resistor (R_F_).

Fig. 5 shows the equivalent electrical model of the force sensor and inverting amplifier circuit for force-to-voltage conversion. When an external force is applied to the force sensor, the force sensor resistance (R_S_) changes from infinity to a specific value depending on the magnitude of the force. The value of R_S_ decreases as the magnitude of the external force increases. When the reference input voltage − V_REF_, a current is generated. Since this current flows into the negative terminal of the OP-amp, its magnitude is equal to the output voltage V_OUT_ divided by the feedback resistor R_F_, according to Kirchhoff’s current law. In this case, the gain of this circuit is V_OUT_/V_REF_ (=R_F_/R_S_), and the overall gain is determined by adjusting R_F_.

#### 5.1.3 Measurement set-up for measuring compressive mechanical property of microstructure

**Fig. 6:**
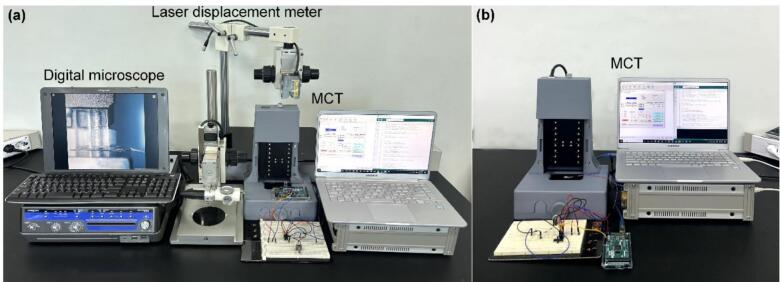
Measurement set-up for evaluating the mechanical properties of microstructures. Validation setup (a), The MCT configuration (b).

Note: The digital microscope and laser displacement meter were employed only for validation purposes, not as integral components of the developed device.

Fig. 6 shows the measurement setup for evaluating the mechanical properties of microstructures in the small-displacement region. The hardware consists of the MCT (comprising a housing-integrated z-stage, laptop, inverting amplifier circuit, and motor controller), a laser displacement meter, and a digital microscope (KH 7700, HIROX, Japan). The assembly of the MCT begins by combining the housing with the z-stage. For displacement control, the linear actuator of the z-stage is connected to the motor controller, and the moving platen, which is the driving component of the z-stage, is aligned with the central axis of the x–y stage. At this point, the force sensor is fixed at the center of the x–y stage. The digital microscope is positioned to observe the specimen on the x–y stage, while the laser displacement meter is arranged so that the aluminum reflector attached to a slide glass on top of the moving platen of the z-stage is aligned perpendicularly to the laser beam.

## 6 Operation instructions

### 6.1 Initialization

Using the Arduino control software (Arduino IDE), upload the force measurement code provided in the source file repository. Ensure that no load is applied to the force sensor, and in the no-load state, verify that the output voltage is initialized to zero. In addition, set the zero position of the z-stage by using the home search function of the MR220A motor control software connected to the linear actuator. Subsequently, the initial calibration of the force sensor was conducted using a 12 g standard weight (corresponding to 0.12 N based on gravitational acceleration, g = 9.8 m/s^2^). The weight was placed on the sensor to obtain the output voltage, which was converted into force via the Arduino system and compared with the 0.12 N reference value, confirming the calibration accuracy of the MCT.

### 6.2 Performing a compression test

**Fig. 7:**
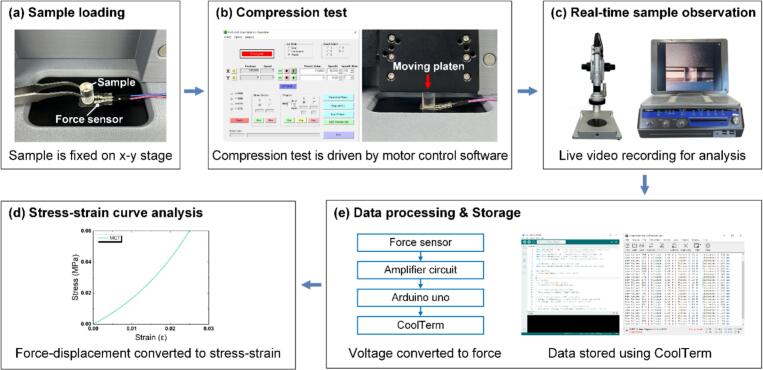
Workflow of compression testing of soft materials using the proposed MCT.

Fig. 7 shows the overall operating procedure of the compression test. To perform a compression test using the overall system, the specimen is first fixed at the center of the x–y stage directly below the z-stage. Using the motor control software, the moving platen is lowered at a specified speed and distance (e.g., 1 μm/s, 500 μm) until it contacts the sample surface, thereby continuously applying a load. During this process, the output voltage signal passes through the inverting amplifier circuit and is converted into force data by the Arduino. The converted force data are transmitted to the Arduino IDE software and stored using CoolTerm software. When the desired displacement or force is reached, the compression test is terminated. The moving platen is then raised, the sample is removed, and the force–displacement data simultaneously stored in CoolTerm are obtained and, if necessary, converted into a stress–strain curve. In addition, surface micro-deformation and fracture during the compression test can be observed using a digital microscope, with videos and images recorded in real time for subsequent analysis.

### 6.3 Safety considerations

During system operation, caution must be exercised to prevent injury and equipment damage. When the motor is running, it may pose a risk of entrapment. therefore, hands or other body parts must not be placed near the z-stage. Although the system operates on a low-voltage DC power supply, all circuit connections must be kept secure, and a safe 5 V power source, such as a USB port or a regulated power supply, should be used to prevent short circuits. In addition, the device must be installed in a stable position to prevent shaking or tipping during testing.

## 7 Validation and characterization

### 7.1 Characterization

The compression testing system integrates a force sensor, an Arduino-based data acquisition system, a precision-controlled z-stage, and both a laser displacement meter and a digital microscope for validation purposes. The device performs accurate force measurements during compression tests. The motor control enables micron-level displacement adjustments. Key performance aspects, including force measurement accuracy, displacement resolution, and test repeatability, were quantitatively validated. The detailed procedures and results are presented in the following sections.

### 7.2 Validation

#### 7.2.1 Validation of the vertical displacement of the z-axis linear actuator

**Fig. 8:**
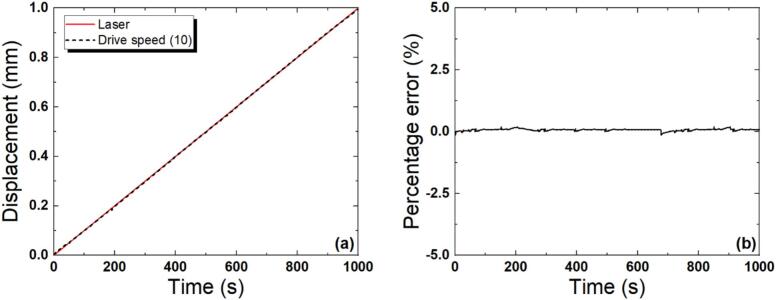
Displacement measured using the laser displacement meter and that obtained from the MR220A software (a), and their relative error (b).

Fig. 8 shows the displacements measured by the laser meter and those obtained from the MR220A software, along with the relative error. When the vertical movement speed of the z-stage (the z-axis linear actuator) is input using the MR220A software (sampling rate ≈ 1 ms, 1 kHz), the linear actuator moves vertically. At this time, to ascertain the precision of the actual displacement in terms of the set input signal, the vertical displacement was measured using the laser meter shown in Fig. 4, and the set actuator and actual displacements were directly compared. A slide glass with an aluminum film on the top of the linear actuator was fixed to ensure laser point reflection. The resolution of the linear actuator was 0.002 mm/pulse (full-step)**,** and, based on this, one pulse was applied every 2 s. The actual displacement was verified by comparison with that of the laser meter, and the vertical displacement measured using that meter was obtained using the LK-Navigator software (maximum sampling rate = 50 kHz). The pulse output signal corresponding to the motor input of the linear actuator was obtained through the MR220A software, and the two displacements were compared to determine the drive speed setting and resolution. The experiment was repeated 10 times and average values were calculated. The actual and set movement speeds were almost identical, with an average error rate of ± 0.1 %, a variance of 0.41, and a standard deviation of ± 0.17.

#### 7.2.2 Optimization of R_F_

**Fig. 9:**
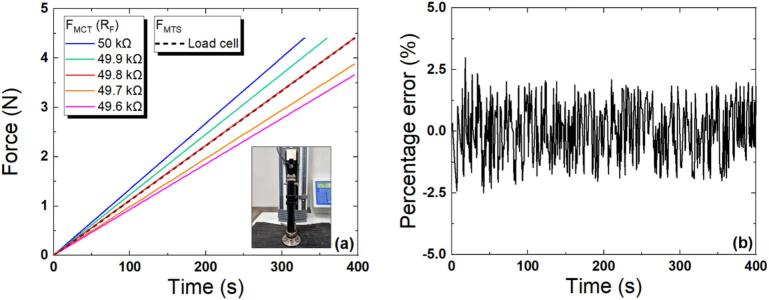
Temporal response of forces measured with MCT and MTS (a) and the percentage error at R_F_ was 49.8 kΩ (b).

Initially, a baseline experiment was conducted to determine the R_F_ value. With the drive voltage fixed at − 5 V, the maximum measurable force was 3.9 N when R_F_ was 100 kΩ. Subsequently, when R_F_ was reduced to approximately 50 kΩ, a maximum measurable force of 4.4 N was obtained, as specified in the force sensor datasheet [97]. To ensure the accuracy and reliability of compression testing, optimization of the R_F_ value, which serves as the gain when adjusting the magnitude of the output voltage, is required. Such optimization was performed via comparison of the MTS and MCT. First, as shown in Fig. 9(a), a force sensor was attached to the specimen-loading part of the MTS, and time-dependent force data were measured by setting the maximum force in the MTS settings to 4.4 N (the maximum measured by the force sensor). At this time, the load cell mounted on the MTS equipment had a capacity of 100 N. The measured force (F_MCT_) stored in the MCT for various R_F_ values was directly compared with the MTS data (F_MTS_), and the percentage error was calculated as shown in Equation (1). All measurements were repeated a total of 10 times, and the average values were calculated and compared. As shown in Fig. 9(b), as the resistance of R_F_ increases, the magnitude of the voltage or force also rises, and vice versa. When the resistance was 49.8 kΩ, the error was small, at ± 2.0 %, with a variance of 1.42 and a standard deviation of ± 1.19. Therefore, the optimal R_F_ value for the compression test was determined to be 49.8 kΩ.

(1) $$\mathrm{Percentage}\;\mathrm{error}\left(\%\right)=\frac{\left(F_{\mathrm{MCT}}-F_{\mathrm{MTS}}\right)}{F_{\mathrm{MTS}}}\times 100$$

#### 7.2.3 Applicability of miniature specimens

The mechanical properties of PDMS are sensitive to the mixing ratio of the base polymer and the curing agent, and the curing time [54], [55], [98], [99], [100], [101]. Although various attempts have been made to identify the mechanical properties, most studies used lab-fabricated specimens rather than specimens prepared using the ASTM standards [102], [103], [104].

**Fig. 10:**
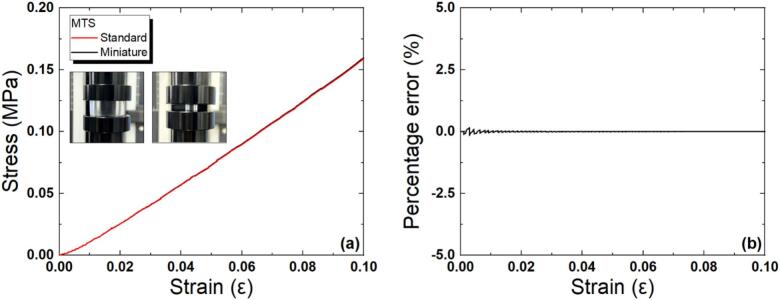
Measured stress–strain curves of the two specimens (a) and the percentage error (b).

In this study, standard specimens for compression testing in the small-displacement regime of PDMS were fabricated and evaluated. One set of specimens was prepared with consideration of the measurement area of the MCT force-sensing unit. These were miniatures of the standard specimen. The compression test results were compared to verify the applicability of the miniature specimens. Finally, the Young’s modulus of PDMS was obtained via compression tests using the MCT. The two types of PDMS specimens were as follows. The standard specimen had the specifications of ASTM D575-91 (diameter: 28.6 ± 0.1 mm, height: 12.5 ± 0.5 mm). For the miniature specimen, the diameter of the circular pressure measurement area of the force sensor was considered, and the specimen diameter was set to 9.53 mm and the height to 4.20 mm. This specimen was 1/3 the size of the standard specimen. For specimen fabrication, two types of molds were produced using a 3D printer (Objet30 Prime, Stratasys). Liquid PDMS was prepared by mixing the base polymer and curing agent at a 10:1 ratio, followed by pouring into the mold, curing at room temperature for more than 1 day, and then demolding. Fig. 10 shows the measured stress–strain curves of the two specimens. The compression tests of the standard and miniature specimens were performed using the same equipment (the MTS). The cross-head speed of the MTS was set to 5 mm/min with reference to the literature values [105]. The compression characteristics of the two specimens were very similar, with an error of + 0.05 %. This demonstrated that the miniature and standard specimens exhibited similar compression behavior within the measurement range up to a strain of 0.1, and that the miniature specimens could also be used for compression tests in the small-displacement region.

#### 7.2.4 Validation of MCT efficiency

**Fig. 11:**
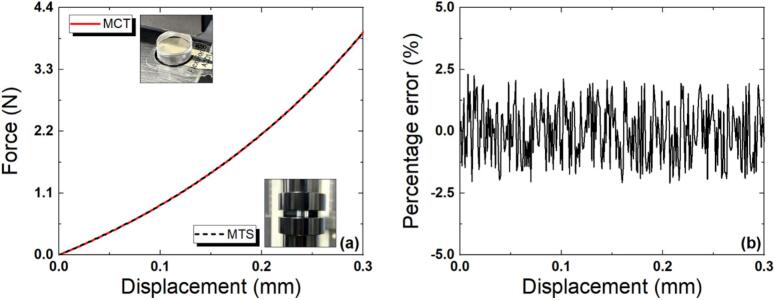
Measured force-displacements of the miniature specimen obtained using the MCT and MTS (a) and the percentage error when R_F_ was 49.8 kΩ (b).

Fig. 11 shows the force–displacement characteristic graph for the miniature specimen when R_F_ was 49.8 kΩ. In the previous section ‘*7.2.3 Applicability of miniature specimens’* the compression tests on two types of specimens were performed using the same equipment. In this section, compression tests were performed on the small specimen using both the MCT and a commercial MTS, and the efficiency of the MCT force-sensing unit was evaluated by comparing the results. As confirmed in *7.2.2. Optimization of R_F_*, when R_F_ was 49.8 kΩ, the measurement results of the two devices were similar, with an error of ± 2.0 %. These results show that the measured mechanical properties of the miniature specimen were very similar when using either test platform, and that the miniature model and the MCT equipment can be employed for compression testing in a small-displacement or small-force regime.

#### 7.2.5 Young’s modulus estimation

**Fig. 12:**
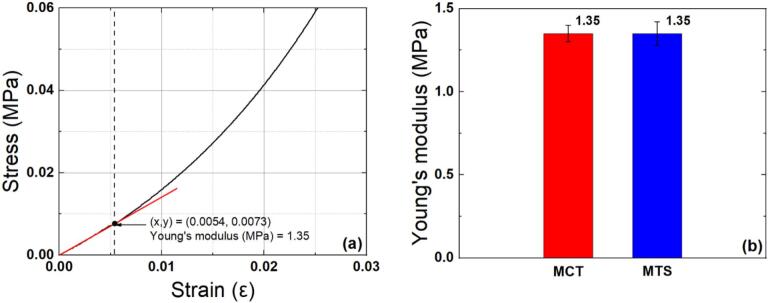
Stress–strain curve of PDMS using MCT (a) and comparison of Young’s modulus by the MCT and MTS (b).

Fig. 12a shows the conversion of the force–displacement curve obtained via MCT compression testing of the miniature PDMS specimen into a stress–strain curve. The Young’s modulus was estimated from the initial slope [106], [107]. The measured Young’s modulus was approximately 1.35 MPa, similar to that of the standard specimen prepared in this study, and to the previously reported Young’s modulus (1.32 MPa) measured using a universal testing machine (Avery Denison, T42U) [55]. The error was + 2.2 %.

Fig. 12b illustrates both the mean and standard deviation (SD) of the measured Young’s modulus values for the two systems (MCT and MTS) together. All experiments were performed three times. SD was calculated from each dataset and reflected in the error bars of the bar graph. The results showed an SD of 0.05 MPa (approximately ± 3.7 % of the mean) for MCT and an SD of 0.07 MPa (approximately ± 5.2 % of the mean) for MTS.

**Summary**.

## 8 Conclusion

This study developed a compact, low-cost micro-compression testing device (MCT) to precisely evaluate the compressive mechanical properties of microstructures in the small-displacement/low-load regime, addressing the lack of laboratory-level instrumentation where conventional commercial testers are impractical or cost-prohibitive. The MCT integrates a high-precision z-stage with an Arduino-based short-stroke force sensor and achieves displacement and force resolutions of 1 µm and 0.01 N, respectively. Cross-validation against commercial testers demonstrated force–displacement agreement within ± 2.0 %, and comparative compression tests verified that the difference between standard PDMS specimens and miniature specimens remained within ± 0.05 %. Using the MCT, the Young’s modulus of PDMS in the small-strain region was measured to be 1.35 MPa, deviating by + 2.2 % from literature values, confirming measurement accuracy and reproducibility at the miniature scale. These results show that the proposed MCT delivers reliable precision while substantially reducing system complexity and cost, thereby enabling routine characterization of microstructures under small-force and small-displacement conditions in standard laboratories.

The platform is thus well-suited for material screening, device prototyping, and educational use where accessible, accurate, and economical compression testing is required.

*Key aspects of the system*.

• The device features a customizable design with a modular structure, allowing adaptation to various micro-scale compression testing applications.
• The cost-effective system (total cost < USD 1,500) is built with open-source electronics and a 3D-printed housing, making it substantially less expensive than commercial testing machines.
• The device ensures high resolution in terms of both displacement and force, enabling precise measurements in the small-strain region.
• The device is compact and lightweight because it is embedded in a 3D-printed housing, enabling convenient use in standard laboratories that lack bulky equipment.
• The hardware and software employ open-source components such as Arduino, CoolTerm, and MR220A, ensuring reproducibility and allowing further modifications by other researchers.

## CRediT authorship contribution statement

**Jaehyeong Kim:** Writing – original draft, Methodology, Investigation, Conceptualization. **Sangjun Pyo:** Writing – original draft, Validation, Formal analysis, Data curation. **Hyerin Ahn:** Writing – original draft, Visualization, Software, Formal analysis. **Ok Chan Jeong:** Writing – review & editing, Supervision, Project administration, Funding acquisition.

## Declaration of competing interest

The authors declare that they have no known competing financial interests or personal relationships that could have appeared to influence the work reported in this paper.
